# Paroxysmal Supraventricular Tachycardia With Wolff-Parkinson-White (WPW) Syndrome: A Therapeutic Dilemma During Pregnancy

**DOI:** 10.7759/cureus.105800

**Published:** 2026-03-24

**Authors:** Rajeev Bharadwaj, Eshwar Raipalle, Suman Kalita, Barkha Jain, Bhupen Barman

**Affiliations:** 1 Cardiology, All India Institute of Medical Sciences, Guwahati, Guwahati, IND; 2 General Medicine, All India Institute of Medical Sciences, Guwahati, Guwahati, IND; 3 Internal Medicine, All India Institute of Medical Sciences, Guwahati, Guwahati, IND; 4 Internal Medicine, North Eastern Indira Gandhi Regional Institute of Health and Medical Sciences, Shillong, IND

**Keywords:** arrhythmia, cardiac sudden death, pregnancy, supraventricular tachycardia, wolf-parkinson-white syndrome

## Abstract

Arrhythmias are among the most common cardiac complications during pregnancy, occurring in women with or without underlying structural heart disease. Early recognition and timely management are crucial to achieving the best possible maternal and fetal outcomes. Supraventricular arrhythmias are particularly frequent during pregnancy. Wolff-Parkinson-White (WPW) syndrome is a rare pre-excitation disorder characterized by the presence of an accessory pathway and can occasionally lead to life-threatening arrhythmias. The exact prevalence of WPW syndrome predisposing to supraventricular tachycardia (SVT) in pregnancy is not known. We present a case of a 28-year-old woman in her second trimester of pregnancy presenting with sudden-onset palpitations. She was hemodynamically stable on presentation, and her electrocardiogram (ECG) recording demonstrated SVT with a heart rate of 226 beats per minute. After a failure of vagal maneuvers, she was successfully treated with intravenous adenosine. Her subsequent ECG was consistent with WPW syndrome with delta waves. This case highlights the complexities in managing a case of SVT in pregnancy with WPW syndrome during its acute phase and follow-up. With pregnancy being a risk factor for an arrhythmogenic state, the presence of an accessory pathway may further increase the risk of fatal arrhythmia. Management should be approached keeping both maternal and fetal outcomes in perspective.

## Introduction

Pregnancy is a proarrhythmogenic state, increasing the risk of both new-onset and recurrent arrhythmias [1]. This is attributed to the fluctuations in hemodynamic, autonomic, and hormonal effects leading to an increase in circulating levels of catecholamines and intravascular volume, resulting in increased ventricular end-diastolic volume and atrial stretch, altogether contributing to increased risks of arrhythmias [1]. In women of reproductive age, paroxysmal supraventricular tachycardia (PSVT) is the most commonly encountered arrhythmia [2]. Supraventricular tachycardia (SVT) also represents the most frequent tachyarrhythmia during pregnancy, with a prevalence of 24 per 100,000 hospital admissions, and exacerbation occurs in about 20% of women with pre-existing SVT [3]. Wolff-Parkinson-White (WPW) syndrome is a congenital cardiac pre-excitation syndrome and can result in symptomatic and life-threatening arrhythmias. The prevalence of WPW is estimated at 0.1% to 0.3%, with an annual arrhythmia progression rate of 1% to 2% and a sudden death risk of approximately 0.25% per year or 3% to 4% over a lifetime in symptomatic patients [4]. The exact prevalence of WPW syndrome in pregnancy is not known; however, some reports suggest that pregnancy may increase the risk of tachyarrhythmia in patients with the presence of asymptomatic pre-excitation, potentially leading to adverse maternal and fetal outcomes [5]. Therefore, prompt recognition and appropriate management of arrhythmias in pregnant patients with WPW syndrome are essential to ensure optimal clinical outcomes.

## Case presentation

**Figure 1:**
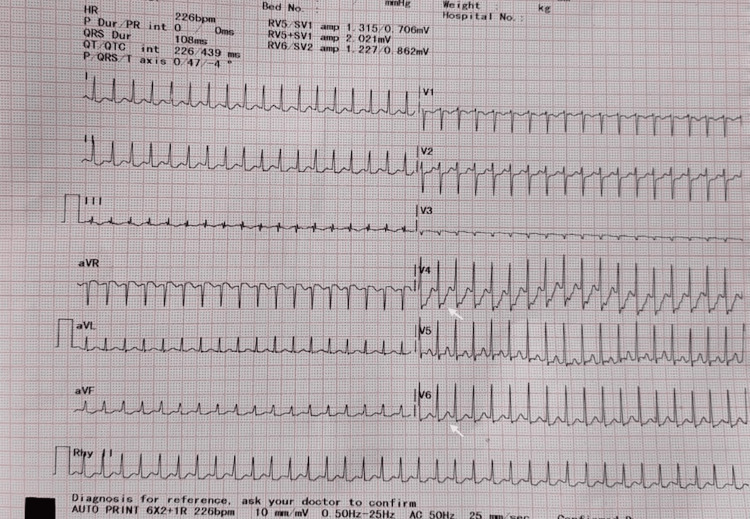
Supraventricular tachycardia. Twelve-lead ECG showing supraventricular tachycardia. The tracing shows regular narrow complex tachycardia with a ventricular rate of 226 beats per minute. Note the retrograde P waves occurring immediately after the QRS complexes as depicted by the white arrows. ECG: electrocardiogram

A 28-year-old female patient with 23 weeks of gestation presented to the emergency department with a history of sudden-onset palpitation and shortness of breath for one day. She denies associated chest pain or syncope. She gave a history of a similar episode in her first pregnancy seven years ago. She recalls hospital admission for the same and was prescribed oral medications. She did not continue any medications after her first pregnancy and occasionally had symptoms of palpitations over the years but never consulted a doctor. On presentation, she had a pulse rate of 215 beats/min and blood pressure of 122/60 mmHg, with other hemodynamic parameters within normal limits. Her cardiovascular and pulmonary examinations were otherwise within normal limits. Her 12-lead electrocardiography (ECG) revealed regular narrow complex tachycardia consistent with SVT, most likely atrioventricular re-entrant tachycardia (AVRT) (Figure 1).

**Figure 2:**
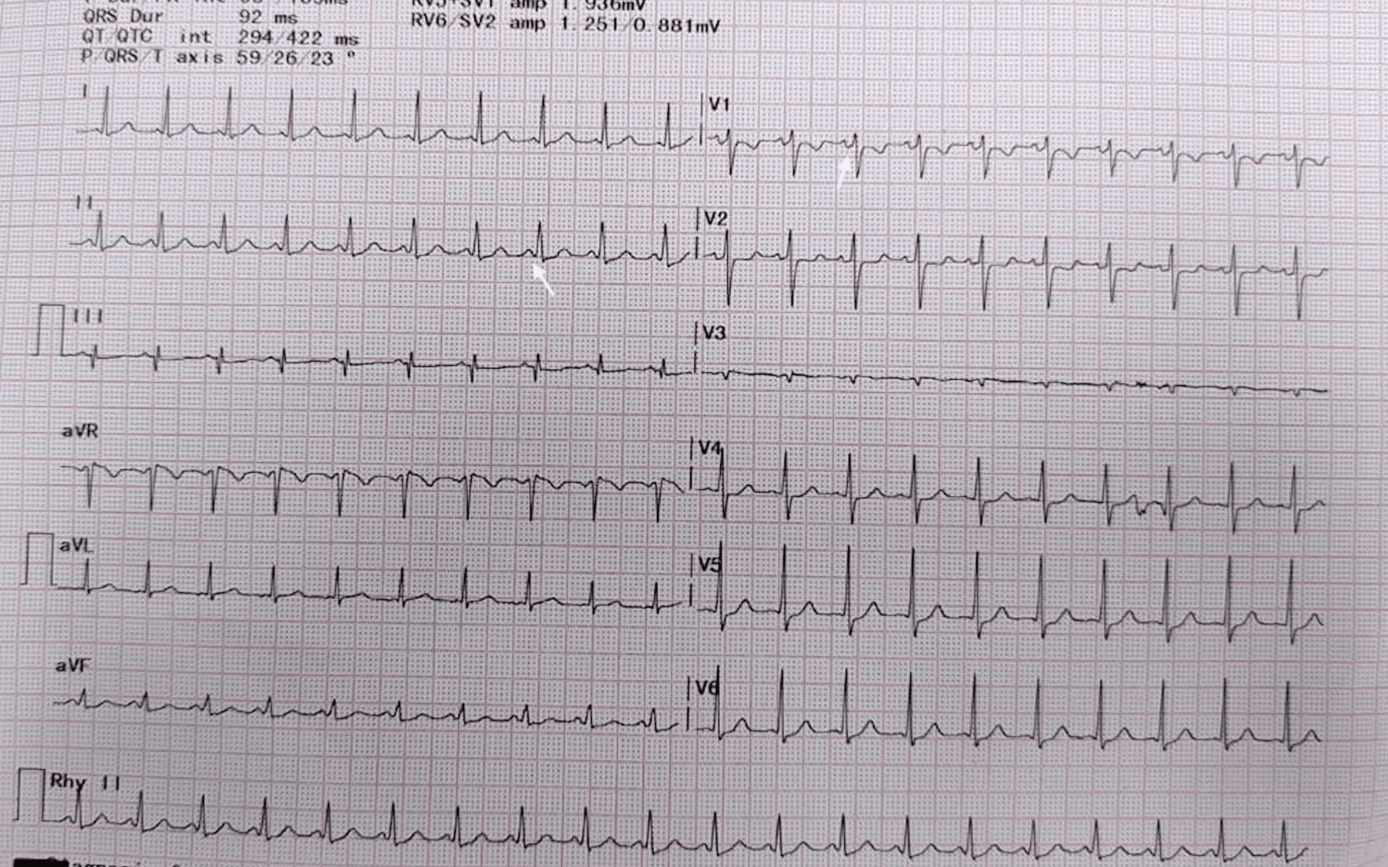
Post-pharmacologic termination ECG with delta waves. Twelve-lead ECG recording after 6 mg of intravenous adenosine showing sinus rhythm at a rate of 124 beats per minute with a short PR interval and slurred QRS upstroke (delta wave) in leads I, II, aVF, V4, V5, and V6 as denoted by white arrows. ECG: electrocardiogram

A trial of vagal maneuver like carotid massage and Valsalva failed to correct the arrhythmia. Injection of adenosine 6 mg intravenously was given by rapid bolus, and the heart rate slowed to 124 beats per minute with a short PR interval of 109 ms and a slurred upstroke in the initial portion of the QRS complex (delta wave) (Figure 2). All routine blood investigations, including complete blood count, thyroid profile, liver enzymes, and electrolytes, are listed in Table 1. Bedside two-dimensional (2D) echocardiographic screening revealed no structural abnormality. Close monitoring of the fetal heart rate was continued during the episode of tachyarrhythmia and after pharmacological termination.

**Table 1:** The patient’s laboratory parameters alongside the reference range. Routine blood test (routine blood examination, renal function, liver function, thyroid function, and NT proBNP levels) performed in a patient alongside the reference values for the same. NT proBNP: N-terminal pro-B-type natriuretic peptide; TSH: thyroid-stimulating hormone

Parameter	Patient value	Reference range
Hemoglobin	10.2 g/dL	13-17 g/dL
Total WBC count	8.73 x 10^9^/L	4-10 x 10^9^/L
Differential cell count	N_72_ L_26_ M_2_ E_0_ B_0_	N_40-80_ L_20-40_ M_2-10_ E_1-6_ B_0-2_
Platelet count	135 x 10^9^/L	150-240 x 10^9^/L
Blood urea	36 mg/dL	19.26-42.8 mg/dL
Serum creatinine	0.8 mg/dL	0.66-1.25 mg/dL
Total protein	6.2 g/dL	6.3-8.2 g/dL
Serum albumin	3.2 g/dL	3.5-5 g/dL
Serum globulin	3.0 g/dL	2.8-3.2 g/dL
Serum total bilirubin	1.5 mg/dL	0.2-1.3 mg/dL
Indirect bilirubin	1.0 mg/dL	0.1-1.1 mg/dL
Direct bilirubin	0.5 mg/dL	0-0.3 mg/dL
Alanine aminotransferase	27 U/L	<50 U/L
Aspartate aminotransferase	48 U/L	17-59 U/L
Alkaline phosphatase	138 U/L	38-126 U/L
Gamma-glutamyl transferase	86 U/L	15-73 U/L
Serum sodium	138 mmol/L	137-145 mmol/L
Serum potassium	4.2 mmol/L	3.5-5.5 mmol/L
Serum calcium	8.8 mg/dL	8.4-10.2 mg/dL
Serum magnesium	2.3 mg/dL	1.6-2.3 mg/dL
Serum NT proBNP	1,420 pg/mL	<300 pg/mL
Serum TSH	2.35 mIU/L	0.40-4.05 mIU/L
Serum free T_3_	2.89 pg/mL	0.40-4.05 pg/L
Serum free T_4_	1.01 ng/dL	0.78-2.19 ng/dL

## Discussion

SVT during pregnancy can pose a clinical challenge because of the potential for hemodynamic instability and maternal-fetal circulatory compromise. The impact of pregnancy on the first onset of SVT remains debated. In a study of 38 patients with PSVT who had prior pregnancies, 34% experienced their first episode during pregnancy [6]. Recurrence is also common, with approximately 50% of women experiencing PSVT again during pregnancy. The risk is higher in women with complex congenital heart disease, which ranges from 0.8% in atrial septal defect to 15.6% in transposition of the great arteries [7].

The most common underlying mechanism of SVT is re-entry. Among these, atrioventricular nodal re-entrant tachycardia (AVNRT) accounts for approximately 60% of cases, while AVRT contributes about 30% [8]. In WPW syndrome, conduction through the accessory pathway is manifested in the ECG with a short PR interval and a widened QRS with an initial slurring upstroke (delta wave) in the sinus rhythm [9]. Majority of patients with an accessory pathway can however remain asymptomatic. Although the exact prevalence of WPW syndrome during pregnancy is not well established, pregnancy may trigger tachyarrhythmias in those with asymptomatic pre-excitation.

Management of SVT in pregnancy requires careful consideration, as most antiarrhythmic medications cross the placenta. Drug therapy is best avoided during the first trimester unless necessary, with careful assessment of the risk-benefit ratios. Adenosine and verapamil are effective in terminating approximately 90% of acute SVT episodes by prolonging atrioventricular nodal refractory periods. Beta-blockers are generally preferred for WPW-related tachycardias but carry risks such as fetal bradycardia, hypoglycemia, intrauterine growth restriction, and neonatal apnea. In patients of atrial fibrillation with pre-excitation, digoxin and calcium channel blockers may, however, enhance conduction across accessory pathways, thereby increasing the risk of fatal arrhythmia [10].

In hemodynamically stable patients, vagal maneuvers should be the first-line intervention. If ineffective, intravenous adenosine (6-18 mg) is the drug of choice, terminating about 90% of PSVT cases [10]. In instances of hemodynamic compromise or persistent arrhythmia, emergency synchronized electrical cardioversion (150-200 J) is recommended. Electrical cardioversion can be safely carried out in pregnancy, with uninterrupted fetal monitoring before and after the procedure [11].

For prophylaxis in symptomatic WPW during pregnancy, beta-blockers may be used with close fetal monitoring. Radiofrequency ablation is generally avoided during pregnancy due to fetal radiation exposure risks. However, in selected drug-refractory cases, catheter ablation using advanced technologies that reduce fluoroscopy risks, like three-dimensional (3D) mapping and intracardiac echocardiography (ICE), can be performed in specialized centers [12]. Current guidelines, however, recommend postponing elective ablation until after delivery [13].

Limitations of the study

The major limitation of this study includes the lack of follow-up to assess whether beta-blocker treatment led to symptom control and to evaluate fetal outcome. Additionally, the unavailability of long-duration ECG monitoring prevented calculation of the shortest pre-excited RR interval, which is important for prognostic assessment and management in WPW syndrome.

## Conclusions

An arrhythmogenic state during pregnancy may exacerbate supraventricular tachyarrhythmia. In patients with underlying WPW syndrome, the presence of an accessory pathway further increases the risk of potentially life-threatening arrhythmias. This case highlights the successful acute management of SVT in a pregnant patient with previously unrecognized WPW syndrome. The importance of early recognition and appropriate stepwise management remains crucial. Adenosine remains a reliable first-line agent for acute termination even in pregnancy, after an appropriate trial of vagal maneuvers. A multidisciplinary approach is essential to ensure optimal maternal and fetal outcomes. Long-term management, including consideration of definitive therapies such as catheter ablation, should be planned in the postpartum period, particularly in patients with persistent symptoms or those at higher risk of recurrence.
